# Evaluation of Khat (Catha edulis) Use as a Risk Factor of Cancer: A Systematic Review (Chong et al., 2020)

**DOI:** 10.31557/APJCP.2020.21.8.2181

**Published:** 2020-08

**Authors:** Sadeq A. Al-Maweri, Walid A. Al-Soneidar, Khalil W. AlQahtani

**Affiliations:** 1 *Department of Oral Medicine and Diagnostic Sciences, Al - Farabi Colleges for Dentistry and Nursing, Riyadh, Saudi Arabia. *; 2 *Department of Oral Medicine, Oral Pathology and Oral Radiology, Faculty of Dentistry, Sana’a University, Yemen. *; 3 *Department of Epidemiology, Biostatistics and Occupational Health, Faculty of Medicine, McGill University, Canada. *; 4 *Al Farabi Colleges for Dentistry and Nursing, Riyadh, Saudi Arabia. *

**Dear Editor**

We have read with interest the recently published systematic review entitled “Evaluation of Khat (Catha edulis) Use as a Risk Factor of Cancer: A Systematic Review” by Chong and colleagues (Chong et al., 2020). We would like to congratulate the authors for this interesting review article, and make some comments.

Khat chewing has been associated with numerous oral/dental disorders such as keratotic white lesions and periodontal diseases (Al-Maweri et al., 2014; Dhaifullah et al., 2015; Kalakonda et al., 2017; Al-Maweri et al., 2018a; Al-Maweri et al., 2018b). Additionally, some reports linked Khat to oral cancer (Soufi et al., 1991; Awang et al., 1993). However, the claimed association between oral cancer and khat was based on anecdotal reports and cross-sectional studies that did not account for important confounding factors, especially tobacco use. Tobacco use (including smoking and smokeless tobacco use) is a well-known risk factor for oral cancer (Warnakulasuriya, 2020). It is important to note that most khat chewers are current smokers and some use smokeless tobacco (locally known as shammah), making it difficult to disentangle these substances’ carcinogenic effect from that of khat (Al-Maweri et al., 2014; Al-Tayar et al., 2015; Al-Maweri et al., 2018a). Contrary to the aforementioned reports that linked khat to oral cancer, one recent case-control study investigating risk factors of oral cancer in Yemen found that khat chewing was not associated with oral cancer, and that shammah was the only risk factor associated with oral cancer, with odds ratio of 12.6 (95% CI 3.3- 48.2) and 39 (95% CI 14-105) for ex-users and current shammah users, respectively (Nasher et al., 2014). In another case-control study of oral cancer in Jazan, Saudi Arabia, the authors did not find any association between khat use and the risk of oral cancer (Quadri et al., 2015). In fact, the latter study reported that khat use reduced the risk of oral cancer when used concurrently with shammah (Quadri et al., 2015). This highlights the importance of studies with enough power to investigate the presence of biologic interaction between khat and tobacco, which could be synergistic or antagonistic in interactive effect. Such results further attenuate the already weak evidence regarding the claimed relationship between khat and oral cancer. As mentioned earlier, the potential association between khat and oral cancer is still questionable and lacks concrete evidence. Because clinical trials are impossible to conduct for ethical and logistic reasons, large-scale cohort studies are required to explore the potential association between khat chewing and oral cancer. 

Another important point included in the review is the potential association between khat use and oral premalignant lesions. The review indicated that “khat can cause premalignant lesions”; such conclusion needs further clarification. It should be noted that the term “premalignant/precancerous lesions” (lately replaced by “potentially malignant lesions” term) refers to a group of lesions that may precede the development of oral cancer (i.e., lesions that have the potential to transform into oral cancer) (Warnakulasuriya, 2020). This includes oral leukoplakia, erythroplakia, oral submucous fibrosis, oral lichen planus, hyperplastic candidiasis, smokeless tobacco-induced lesions, among others (Warnakulasuriya, 2020). However, to date, there is no any evidence that khat-induced lesions has the potential to transform into oral cancer, and hence such lesions are not classified as premalignant lesions. Current evidence suggests that Khat-induced white lesions are benign keratotic lesions, which occur in close proximity to the site of khat bolus, mainly on the buccal mucosa of the chewing side, and usually disappear in a few weeks after quitting the habit (Al-Maweri et al., 2018a). These lesions are attributed to the continuous mechanical friction during chewing and to the drying effects of khat (Al-Maweri et al., 2018a). The association between keratotic white lesions and khat chewing has been established in the literature (Al-Maweri et al., 2014; Al-Maweri et al., 2018a). However, the potential malignant transformation of these lesions has not been proven. As mentioned above, previous studies did not account for the concurrent use of tobacco, especially smokeless tobacco, when reporting the association of khat chewing with oral premalignant and/ or malignant lesions. Additionally, the evidence obtained in this review was based merely on cross-sectional studies, from which causality cannot be established nor can definitive conclusions be affirmed.

To summarize, the association between khat chewing and oral cancer is still questionable. In a similar fashion, the potential malignant transformation of khat induced-keratotic lesions is still unclear. Hence, future large-scale studies with long follow-up periods that take into consideration all potential confounders are highly recommended to determine the potential association between khat and oral cancer.

## Conflict of interests

The authors declare that they have no any conflict of interests.
